# Circadian rest-activity rhythms during benzodiazepine tapering covered by melatonin versus placebo add-on: data derived from a randomized clinical trial

**DOI:** 10.1186/s12888-016-1062-8

**Published:** 2016-10-13

**Authors:** Lone Baandrup, Ole Bernt Fasmer, Birte Yding Glenthøj, Poul Jørgen Jennum

**Affiliations:** 1 Center for Neuropsychiatric Schizophrenia Research (CNSR) & Center for Clinical Intervention and Neuropsychiatric Schizophrenia Research (CINS), Copenhagen University Hospital, Mental Health Center Glostrup, Mental Health Services-Capital Region of Denmark, DK-Glostrup, Denmark; 2 Division of Psychiatry, Haukeland University Hospital, Bergen, Norway; 3 Department of Clinical Medicine, Section for Psychiatry, Faculty of Medicine and Dentistry, University of Bergen, Bergen, Norway; 4 Rigshospitalet, Danish Center for Sleep Medicine, Department of Clinical Neurophysiology, Center for Healthy Ageing, Faculty of Health Sciences, University of Copenhagen, DK 2600 Glostrup, Denmark

**Keywords:** Schizophrenia, Bipolar disorder, Circadian rhythms, Benzodiazepines, Discontinuation, Withdrawal, Circadian rhythm, Randomized clinical trial

## Abstract

**Background:**

Patients with severe mental illness often suffer from disruptions in circadian rest-activity cycles, which might partly be attributed to ongoing psychopharmacological medication. Benzodiazepines are frequently prescribed for prolonged periods despite recommendations of only short-term usage. Melatonin, a naturally occurring nocturnal hormone, has the potential to stabilize disrupted circadian rhythmicity. Our aim was to investigate how prolonged-release melatonin affects rest-activity patterns in medicated patients with severe mental illness and if benzodiazepine dose reduction is associated with changes in circadian rhythm parameters.

**Method:**

Data were derived from a randomized, double-blinded clinical trial with 24 weeks follow-up. Participants were randomized to add-on treatment with prolonged-release melatonin (2 mg) or matching placebo, and usual benzodiazepine dosage was gradually tapered. Here we report the results of 72 h of actigraphic assessment of activity-rest cycles performed pre and post tapering. Changes in rest-activity rhythm parameters between the melatonin and placebo group were analyzed using the univariate general linear model. Change in activity counts per 6 h, from baseline to follow-up, in the whole sample was analyzed using paired samples *t*-test.

**Results:**

A subsample of 48 patients participated in the actigraphic assessment: 20 in the melatonin group and 28 in the placebo group. Rest-activity cycles varied from regular to highly disrupted. Melatonin significantly increased the interdaily stability and at a trend level decreased the intradaily variability compared with placebo. Benzodiazepine dose reduction was not associated with these circadian rhythm parameters. Activity counts were generally higher after benzodiazepine dose reduction compared with pre tapering, but differences did not reach statistical significance.

**Conclusion:**

Our data suggest melatonin as an aid during benzodiazepine withdrawal for patients distressed by disrupted circadian rest-activity cycles. Benzodiazepine tapering might result in diminished sedentary behavior but further research is needed.

**Trial registration:**

ClinicalTrials NCT01431092, clinicaltrials.gov. Registered 31 August 2011.

## Background

Disturbances in rest-activity cycles in schizophrenia patients compared with healthy controls have been reported in several small-moderate sized clinical studies with sleep recordings lasting from one night to several weeks [1]. Standard nocturnal polysomnographic recordings evaluate sleep stages and sleep architecture, but the obtrusive character of the technique makes it less suitable for investigations across several days to assess circadian rhythmicity. Additionally, polysomnography measures sleep and is thus primarily used during nighttime. In this context, actigraphy has been identified and evaluated as an important tool for assessing circadian rest-activity patterns [2, 3]. In schizophrenia patients, a highly variable pattern of circadian rhythm disturbances have been reported ranging from well-entrained cycles to highly fragmented sleep/wake cycles [4]. In general, motor activity has been found to be markedly reduced and more monotonous in schizophrenia patients compared with controls [1, 5]. It has been suggested that a lack of daily routine activities and a diminished level of zeitgebers might explain the observed rest-activity pattern disruptions observed in schizophrenia patients [6], but recent experimental evidence does not seem to support this view [7]. The contribution of psychopharmacological drugs to the overall picture of circadian rhythm disturbances have only been scarcely investigated, but there is some evidence that antipsychotic drugs influence circadian rhythmicity differentially [8]. In addition to disturbed sleep wake cycles there is evidence of alteration of a number of other circadian parameters in schizophrenia including disturbed regulation of melatonin secretion and core body temperature [9].

Benzodiazepines are frequently prescribed in chronic psychiatric patients to treat complaints of insomnia and anxiety. Due to the side effect burden, benzodiazepines are only recommended for short-term use [10, 11]. However, treatment is often prolonged and subsequent discontinuation may pose a problem due to distressing withdrawal symptoms. Rebound insomnia may occur during withdrawal and via this mechanism, benzodiazepine discontinuation might therefore affect circadian rhythmicity. Melatonin is a naturally occurring hormone secreted at nighttime and implicated in sleep induction and circadian rhythm stabilization. For these reasons, melatonin has theoretical potential as add-on medication for facilitating benzodiazepine discontinuation and observational data seem to suggest a possible role for melatonin in the context of benzodiazepine withdrawal [12, 13].

We aimed to investigate how prolonged-release melatonin affects circadian rhythm parameters in medicated patients with schizophrenia or bipolar disorder and if benzodiazepine dose reduction is associated with changes in motor activity and diurnal rest-activity patterns. We hypothesized that treatment with melatonin would result in a more stable circadian rest-activity pattern compared with placebo. Furthermore, we hypothesized that benzodiazepine dose reduction/discontinuation would be associated with increased motor activity due to lessened sedation and amelioration of apathy and other negative-like symptoms.

## Methods

### Study design and participants

This is a single-center, randomized, double-blinded clinical trial conducted at a university hospital research department in the capital region of Denmark. Participants eligible for the trial were 18 years or above; had a diagnosis of schizophrenia, schizoaffective disorder or bipolar mood disorder (and euthymic at inclusion); were treated with at least one antipsychotic drug and had a daily use of at least one benzodiazepine or benzodiazepine-like drug for a minimum of 3 months; did not present with current violent or aggressive behavior; were not diagnosed with mental retardation, pervasive developmental disorder, dementia, hepatic impairment, terminal illness, severe somatic comorbidity, or epilepsy; were able to understand Danish; and were not allergic to any compounds in the study medication. Fertile women were only included if not pregnant or nursing and if using safe contraceptives throughout the study period.

After baseline investigations, participants were randomized to prolonged-release melatonin (PRM) 2 mg or identical placebo once daily for 24 weeks, and in parallel they were instructed to gradually reduce their daily benzodiazepine usage. Participants were instructed to ingest the study medication approximately 2 h before bedtime with a small meal.

The overall aim of the trial was to investigate if PRM can facilitate discontinuation of long-term benzodiazepine usage in chronic psychiatric patients and these results have been published elsewhere [14] in agreement with CONSORT guidelines. Primary and secondary outcomes are described in the published trial protocol [15]. Here we report the results of actimetric assessments in a subset of the participants during three consecutive days and nights before and after 24 weeks of benzodiazepine tapering. The presented analyses of actimetric data were not planned in details when the trial protocol was published [15].

### Actigraphic assessment and circadian rest-activity cycle measurement

Actigraphy does not measure sleep state and thus cannot differentiate between sleep and sedentary behavior, but it is established as a reliable instrument for evaluating sleep patterns, for studying the effects of treatments to improve sleep, and in the diagnosis of circadian rhythm disorders [2, 3, 16]. A recent review confirmed the clinical usefulness of actigraphy for objective evaluation of sleep habits and circadian rhythm disturbances in psychotic disorders [1].

We measured actimetry for three consecutive days and nights (72 h) to evaluate rest-activity patterns with and without melatonin and before and after benzodiazepine tapering. From the American Academy of Sleep Medicine it has been recommended to use at least three consecutive days of actigraphic recording to obtain reasonable reliability of actigraphic estimates of sleep and 24 h rhythm activity variables [16]. The results of one night polysomnography (at baseline and follow-up), which was performed simultaneously with the first night of actigraphy, will be reported elsewhere. Subjects were asked not to change anything in day or night activities and to continue their usual circadian patterns as uninterrupted as possible while actimetric recordings were done. Recordings were performed from Tuesday to Friday or from Friday to Monday.

We used Actiwatch Spectrum (Philips Respironics) which was continuously worn on the non-dominant arm for a minimum of 72 h. The Actiwatch is a small portable device, the size of a wrist-watch, which uses an accelerometer to detect and log wrist movement (Actiwatch Clinical Implementation Guide; www.actigraphy.com).

Day and night time activity data were logged at 30 s epochs, stored within the Actiware software (version 6.0.0, Respironics, Murrysville, PA, USA), and exported as activity counts per 30 s. We did not use the automatically displayed sleep variables except for wake up time, which was used for alignment of activity counts. Data were then processed to calculate activity counts per hour and activity counts per 6 h. We analyzed activity counts with wake up time (as opposed to clock time) as reference. Wake up time was used as circadian phase marker.

Actigraphic assessed circadian rest-activity cycle parameters included the interdaily stability (IS), the intradaily variability (IV), and the relative amplitude (RA). These non-parametric circadian rhythm parameters have been recommended because they more accurately describe characteristics of disturbances in the rest-activity rhythm and because they are more sensitive to change compared with alternative statistical procedures [17]. The IS quantifies the invariability from day to day, i.e., the predictability of the 24 h rest-activity pattern. This index will be 1 for perfect IS and thus lower values reflect higher variability between the observed days. The IV reflects the fragmentation of the rhythm, i.e., the frequency and extent of transitions between rest and activity. Thus, higher values of IV reflect higher degree of fragmentation and more frequent shifts between rest and activity. The RA is calculated from the ratio of the most active 10 h period to the least active 5 h period in the average 24 h pattern and thus higher values reflect more pronounced differences between periods of rest and activity (typically between day and night time). These non-parametric variables have been used in several studies of circadian rhythm and motor disturbances in schizophrenia [4, 5].

### Statistical methods

We used SPSS version 22 for statistical analyses. All analyses were based on complete cases for actigraphy assessment at baseline and follow-up.

Activity counts were measured in 30 s epochs, converted to 60 s epochs and entered into an excel template for calculation of the three rest-activity rhythm parameters (IS, IV, and RA) across the 72 h of actigraphic assessment at both baseline and follow-up. IS was calculated as the ratio between the variance for the average 24 h pattern around the mean and the overall variance, IV was calculated as the ratio of the mean squares of the difference between all successive hours and the mean squares around the grand mean, and RA was calculated from the most active 10 h period and the least active 5 h period in the average 24 h pattern [17]. Changes in IS, IV, and RA between the PRM and placebo group were analyzed using the univariate general linear model with the outcome measure (24 weeks value) as the dependent variable and the baseline value, intervention group, and benzodiazepine dose reduction (from baseline to end point) as independent variables. In case of non-normal distributions, a non-parametric test (Mann–Whitney *U*-test) was used.

For evaluation of level of activity, the activity counts for the second recorded night and day (from midnight to midnight) at both baseline and follow-up were converted to number per hour and number per 6 h. We chose the second night and day due to the best data quality compared with the first and third night and day. Mean activity count per hour was plotted against time with actigraphy wake up time as reference for the total sample. Change in activity counts per 6 h from baseline to follow-up in the total sample as well per group was analyzed using paired samples *t*-test.

## Results

A subsample of 48 participants were actimetrically evaluated: 20 in the PRM group and 28 in the placebo group. Not all participants in the trial accepted to participate in sleep and actigraphy recordings (not mandatory for trial participation), which led to the unequal numbers of participants in the groups for this outcome. All participants were outpatients. Baseline demographic and clinical characteristics are listed in Table 1. There were no statistically significant differences between the intervention groups.

**Table 1:** Baseline demographic and clinical characteristics

	Prolonged-release melatonin *N =* 20		Placebo *N =* 28	
	*N*	%	*N*	%
Men	11	55.0	18	64.3
Diagnosis				
Paranoid schizophrenia	15	75.0	23	82.1
Non-paranoid schizophrenia	0	0	2	7.1
Schizoaffective disorder	2	10.0	0	0
Bipolar affective disorder	3	15.0	3	10.7
Housing				
Living independently	17	85.0	19	67.8
Supported housing	1	5.0	0	0
Institution	2	10.0	9	32.1
Occupational status				
Employed	0	0	0	0
Financial aid/cash subsidies	0	0	2	7.1
Disability pension	18	90.0	44	91.7
Other	2	10.0	2	4.2
Benzodiazepine treatment				
One drug	15	75.0	22	78.6
Two drugs	5	25.0	6	21.4
Clonazepam	11	55.0	30	62.5
Diazepam	0	0	1	2.0
Oxazepam	5	25.0	9	18.8
Nitrazepam	2	10.0	4	8.3
Lorazepam	0	0	1	2.1
Zopiclone	2	10.0	2	4.2
Zolpidem	0	0	1	3.6
Antipsychotic drug treatment				
One drug	12	60.0	13	46.4
Two drugs	7	35.0	11	39.3
≥ Three drugs	1	5.0	14	14.3
Antidepressant drug treatment				
≥ One drug	14	70.0	14	50.0
Mood stabilizer drug treatment				
≥ One drug	6	30.0	7	25.0
Anticholinergic drug treatment				
One drug	1	5.0	7	25.0
	Mean	SD	Mean	SD
Age, years	47.7	8.2	45.9	10.3
Duration of illness, years	23.3	12.7	18.9	8.1
PANSS total score	61.1	15.2	64.5	12.5
Benzodiazepine treatment duration, years	11.3	9.1	11.6	7.2
Benzodiazepine total dose, mg diazepam equivalents	25.1	24.3	25.5	14.1
Total antipsychotic dose, mg olanzapine equivalents	17.7	11.5	27.6	25.2
IS	0.63	0.10	0.57	0.10
IV	0.74	0.21	0.85	0.26
RA	0.85	0.10	0.87	0.09

*PANSS* positive and negative syndrome scale, *IS* interdaily stability, *IV* intradaily variability, *RA* relative amplitude

No statistically significant differences (*p <* 0.05) between groups at baseline (categorical variables evaluated with chi-square test and continuous variables evaluated with independent *t*-test)

Visual inspection of individual rest-activity cycles showed various degrees of circadian rhythmicity, from regular to highly disrupted rest-activity patterns. We investigated the distribution of IS, IV, and RA at baseline and at follow-up in each intervention group. IS was normally distributed according to visual inspection and the Shapiro-Wilk’s test (*p >* 0.05). The mean IS was 0.61 (95 % CI 0.56 to 0.66) in the PRM group and 0.54 (95 % CI 0.51 to 0.58) in the placebo group at follow-up with a mean difference between the groups of 0.07 (95 % CI 0.01 to 0.13), which was statistically significant (*P =* 0.03), when adjusting for the baseline value in the univariate general linear model. IS was not associated with benzodiazepine dose reduction in the sample as a whole (respective coefficient in the model not statistically significant, *P =* 0.74).

IV and RA were positively skewed and it was not possible to obtain normal distributions after transformation. We therefore analyzed IV and RA non-parametrically (Mann–Whitney *U*-test). When comparing the median value at follow-up for IV (0.81 in the PRM group versus 1.01 in the placebo group) there was a borderline significant difference (*p =* 0.06) with the lowest value (indicating the least diurnal rhythm fragmentation) in the melatonin group, but for RA there was no difference between intervention groups (0.83 in both groups). There was no correlation between benzodiazepine dose reduction and IV (Spearman’s correlation, *r =*−0.052, *p =* 0.79) or RA (Spearman’s correlation, *r =* −0.145, *p =* 0.45) in the whole sample.

**Fig. 1:**
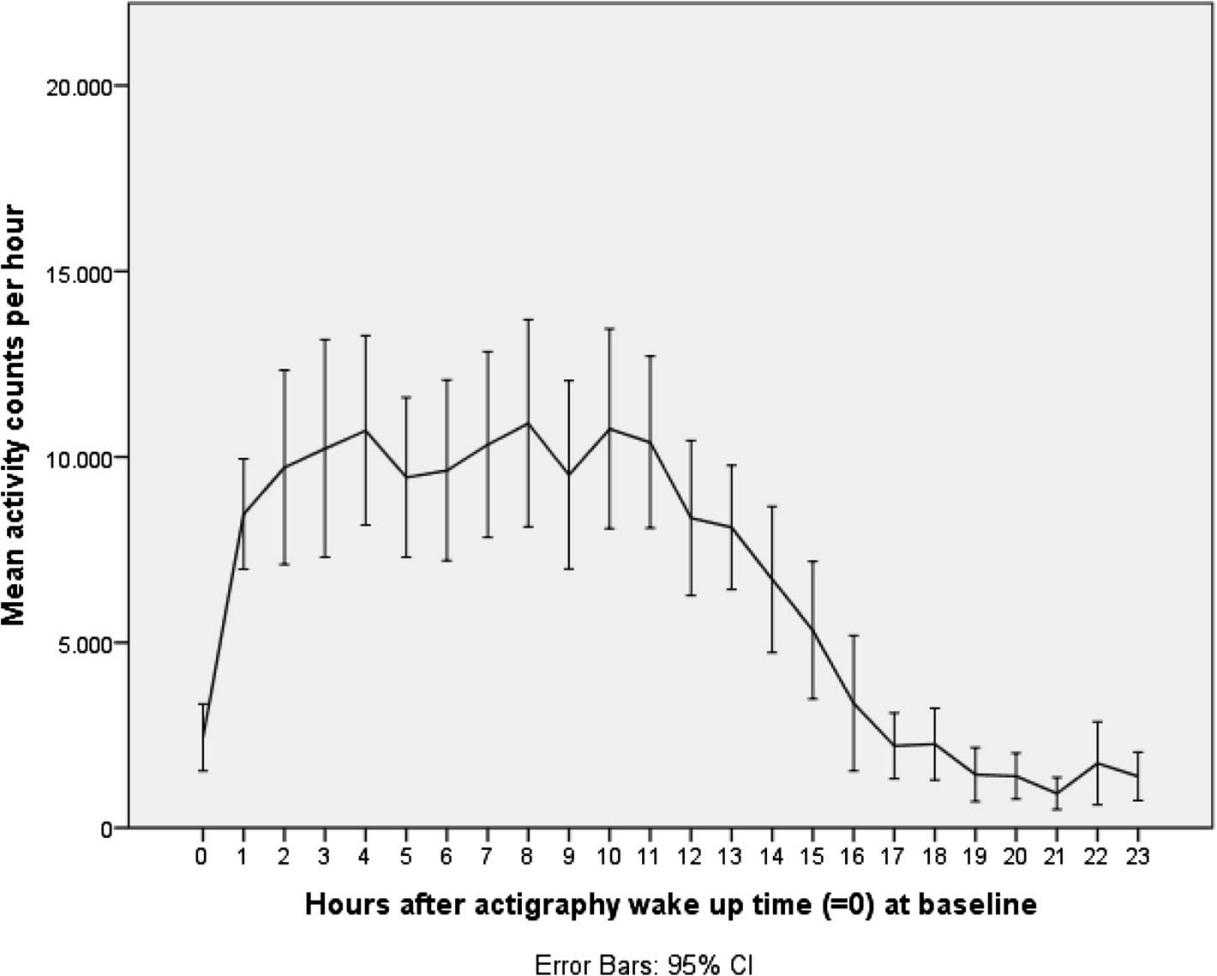
Activity counts at baseline. Legend: Mean activity counts aligned to wake up time (=0) measured the second day and night for the total sample at baseline

**Fig. 2:**
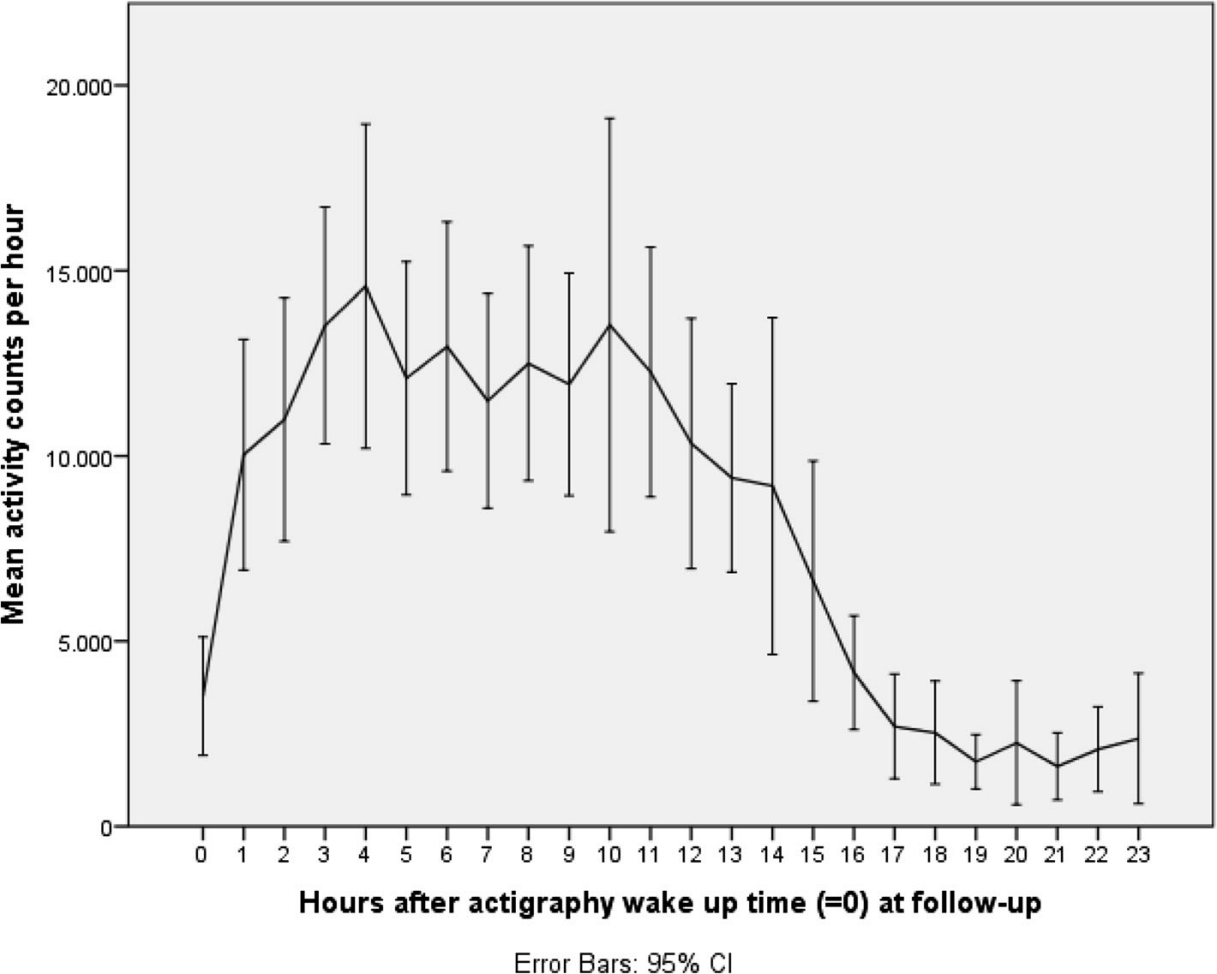
Activity counts at follow-up. Legend: Mean activity counts aligned to wake up time (=0) measured the second day and night for the total sample at follow-up

Figures 1 and 2 illustrate mean activity counts per hour against time after wake up at, respectively, baseline and follow-up. Table 2 presents activity counts per 6 h aligned to wake up time. Activity counts generally increased from baseline to follow-up. However, the differences did not reach statistical significance besides a trend during the first 6 h interval after wake up, e.g., the beginning of the day (*P =* 0.063).

**Table 2:** Activity counts per 6 h (mean ± SD), measured the second night and day in the whole sample (*N =* 30)

	Baseline	Follow-up	Paired samples *t*-test		
			t	df	*P*
1–6 h after wake up time	52240 ± 29594	65176 ± 34731	−1.933	29	0.063
7–12 h after wake up time	59177 ± 33083	74659 ± 40067	−1.655	29	0.109
13–18 h after wake up time	35618 ± 26546	42040 ± 32144	−0.801	29	0.430
19–24 h after wake up time	8423 ± 8817	12299 ± 13271	−1.400	29	0.172

When looking at the intervention groups separately, the results were consistent: there were no statistically significant differences between baseline and follow-up when comparing each of the 6 h intervals (Table 3).

**Table 3:** Activity counts per 6 h (mean ± SD), measured the second night and day in the two intervention groups

	Baseline	Follow-up	Paired samples *t*-test		
	*N =* 11 (Melatonin) *N =* 19 (Placebo)	*N =* 11 (Melatonin) *N =* 19 (Placebo)			
			t	df	*P*
1–6 h after wake up time					
Melatonin	58798 ± 26310	72697 ± 37957	−1.490	10	0.167
Placebo	48444 ± 31383	60822 ± 32990	−1.335	18	0.198
7–12 h after wake up time					
Melatonin	67643 ± 22202	92526 ± 45319	−1.441	10	0.180
Placebo	54276 ± 37688	64314 ± 33724	−0.909	18	0.376
13–18 h after wake up time					
Melatonin	37399 ± 17875	48155 ± 23070	−1.088	10	0.320
Placebo	34578 ± 32279	38500 ± 36509	−0.341	18	0.737
19–24 h after wake up time					
Melatonin	6854 ± 5466	7046 ± 7347	−0.087	10	0.933
Placebo	9332 ± 10308	15341 ± 15071	−1.447	18	0.165

## Discussion

This is the first study investigating changes in rest-activity patterns in a psychiatric population tapered from chronic benzodiazepine use covered by melatonin versus placebo. We found that melatonin stabilized circadian rest-activity rhythmicity by significantly increasing the IS and at a trend level reducing the IV compared with placebo. Benzodiazepine dose reduction was associated with statistically insignificant increases in daytime and nighttime activity counts, and benzodiazepine dose reduction was not correlated with changes in circadian rhythm parameters.

Few studies have investigated circadian rhythmicity objectively in patients with severe mental illness, particularly in relation to clinical trials aiming to evaluate a specific treatment of sleep or circadian rhythm disturbances. Actually, most clinical insomnia trials have excluded participants with psychiatric comorbidity. Melatonin has previously been evaluated in two clinical trials with schizophrenia patients, but not with a specific focus on stabilization of circadian rhythm disturbances. Shamir et al. [18] reported an improvement with melatonin add-on in actimetry-derived sleep efficiency with an effect size of 0.4 in a randomized, blinded, cross-over clinical trial in schizophrenia patients complaining of poor sleep quality and diagnosed with insomnia. However, this was a small trial with only 19 participants and no measure of rest-activity pattern. Kumar and colleagues conducted a randomized, placebo-controlled, double-blind clinical trial investigating the efficacy of 15 days flexibly dosed melatonin in 40 schizophrenia outpatients [19]. Improvement was found in several self-assessed sleep-parameters with the clinically most relevant being an increase in sleep duration, but no objective sleep or activity assessment was performed. In a randomized clinical trial of 83 euthymic bipolar patients, treatment with ramelteon (a melatonin receptor agonist) markedly reduced relapse rate (emergence of depressed or manic episode) throughout the 24-week treatment period, but again objective sleep or activity assessments were lacking [20]. Our findings of circadian stabilization with melatonin treatment should be further investigated, but indicate that melatonin might be a valuable treatment option for patients with severe mental illness distressed by disrupted rest-activity patterns. Supporting this suggestion is experimental evidence indicating severe circadian misalignment in melatonin cycles in a substantial proportion of patients with schizophrenia [7] together with observations of a comprised sleep-promoting action of endogenous melatonin in schizophrenia [21].

The implications of normalized versus disturbed circadian rest-activity patterns has been demonstrated on several levels. Bromundt et al. recently conducted a study of 14 patients with schizophrenia who were actimetrically assessed for 3 weeks and found that participants with a normal rest-activity cycle performed better in frontal lobe function tasks [4]. A similar conclusion was reached in a study of 28 schizophrenia patients undergoing 3 days of actigraphy [22], where patients with more disturbed sleep and less robust circadian rhythms performed more poorly on neuropsychological tests. For patients with bipolar disorder, it has been suggested that disrupted circadian sleep-wake cycles contribute to relapse [23]. However, the direction of the association between circadian disturbances and symptom level needs further exploration and might be bidirectional or, alternatively, reflecting different expressions of the same underlying neuropathological disturbances. Suggested mechanisms of circadian rhythm impairment in schizophrenia include genetic mutations associated with both schizophrenia and circadian regulation [24] and inherent neurotransmitter dysfunctions associated with schizophrenia that also disrupt mechanisms of sleep/wake regulation in the brain stem [25]. A differential role of antipsychotics have been reported [5, 8]*,* but circadian rhythm disruptions are also present in un-medicated patients [26]. Evidence suggests that antipsychotics ameliorate rather than impair sleep disturbances [27, 28], whereas the influence of antipsychotics and other psychotropics on rest-activity cycles has only been scarcely studied [26]. To our knowledge, the influence of benzodiazepine dose reduction/discontinuation on circadian rest-activity cycles has not previously been investigated. Our findings of increased motor activity, albeit not statistically significant, with benzodiazepine dose reduction is interpreted as reflecting a diminished level of sedation due to reduced or discontinued benzodiazepine use. One should be aware that increased motor activity during nighttime might indicate insomnia. However, since the non-significant increase in motor activity was dispersed throughout the day and night it seems more plausible that the finding reflects an increase in spontaneous motor activity.

One limitation of our study is the limited duration of actigraphic recording. We chose the minimum recommended duration of 3 days [17] to increase acceptability, but other evidence points to the fact that a longer period of recording is required to reduce the variability and increase the reliability of obtained measures [18]. Due to logistic reasons, it was not possible to obtain all actimetric recordings during the same days of the week. For some participants recordings were done during weekdays and for other participants recordings were done during the weekend. This might have influenced the results because of different patterns and levels of activity during weekdays compared with weekend days. However, all the participants were unemployed and thus did not report much difference in activity pattern between weekdays and weekend days. Another limitation regarding the evaluation of effect of benzodiazepine dose reduction on the 24 h activity pattern is the lack of a control group continuing usual benzodiazepine consumption, but this was not considered feasible due to the range of side effects associated with continued benzodiazepine usage. The majority of participants in this study were diagnosed with schizophrenia and in the complete case sample (30 patients undergoing actigraphy both at baseline and at follow-up), 75% (25 patients) of the patients had a diagnosis of schizophrenia and 25 % (5 patients) were diagnosed with bipolar disorder. Circadian rhythm disturbances in bipolar patients are most pronounced during episodes of mania or depression, but inter episode disturbances in sleep and circadian rhythmicity also exist and have been reported to more closely resemble sleep disturbances in insomnia patients than in healthy controls [23]. Since sleep and circadian rhythm disturbances are also highly prevalent in patients with schizophrenia [27] and since all bipolar patients, who underwent actigraphic assessment, were euthymic throughout the study period, we found it relevant to analyze the diagnostic categories together. Overall, due to the limited sample size the study had a lack of power to detect more subtle differences between intervention groups. Another obvious limitation is that 18 participants out of 40 did not participate in actigraphic assessment at follow-up, which may have hampered the external validity of the results. This was not necessarily identical with drop out from the overall study aim (benzodiazepine dose reduction), but many of the participants were reluctant to repeat the sleep examinations at follow-up, even if they were given the opportunity of only repeating actigraphy and not the polysomnographic recordings. When analyzing the complete case sample and the sample with sleep-activity recordings only at baseline, no statistically significant differences appeared indicating that the patients not completing the activity recordings at follow-up were not markedly different from the complete cases.

A clear advantage of our study, compared with other studies evaluating circadian rhythm disturbances in severely ill patients, is the randomized controlled design. Most other studies of actimetric evaluation in schizophrenia patients were cross-sectional and without assessing effects of any intervention.

## Conclusions

Add-on treatment with PRM seems to stabilize circadian rest-activity cycles in patients with schizophrenia or bipolar disorder, which is in line with theoretical assumptions of melatonin activity. Benzodiazepine dose reduction might be associated with increased daytime and nighttime motor activity but further research is needed.

## Abbreviations

IS: Interdaily stability
IV: Intradaily variability
PRM: Prolonged-release melatonin
RA: Relative amplitude
